# Sr analyses from only known Scandinavian cremation cemetery in Britain illuminate early Viking journey with horse and dog across the North Sea

**DOI:** 10.1371/journal.pone.0280589

**Published:** 2023-02-01

**Authors:** Tessi Löffelmann, Christophe Snoeck, Julian D. Richards, Lucie J. Johnson, Philippe Claeys, Janet Montgomery

**Affiliations:** 1 Department of Archaeology, University of Durham, Durham, United Kingdom; 2 Department of Chemistry, Research Unit: Analytical, Environmental & Geo-Chemistry, Vrije Universiteit Brussel, AMGC-WE-VUB, Brussels, Belgium; 3 G-Time Laboratory, Université Libre de Bruxelles, Brussels, Belgium; 4 Department of Art Sciences & Archaeology, Maritime Cultures Research Institute, Vrije Universiteit Brussel, Brussels, Belgium; 5 Department of Archaeology, University of York, The King’s Manor, Exhibition Square, York, United Kingdom; University of Padova: Universita degli Studi di Padova, ITALY

## Abstract

The barrow cemetery at Heath Wood, Derbyshire, is the only known Viking cremation cemetery in the British Isles. It dates to the late ninth century and is associated with the over-wintering of the Viking Great Army at nearby Repton in AD 873–4. Only the cremated remains of three humans and of a few animals are still available for research. Using strontium content and isotope ratios of these three people and three animals–a horse, a dog and a possible pig–this paper investigates the individuals’ residential origins. The results demonstrate that strontium isotope ratios of one of the adults and the non-adult are compatible with a local origin, while the other adult and all three animals are not. In conjunction with the archaeological context, the strontium isotope ratios indicate that these individuals most likely originated from the area of the Baltic Shield–and that they died soon after arrival in Britain. This discovery constitutes the first solid scientific evidence that Scandinavians crossed the North Sea with horses, dogs and other animals as early as the ninth century AD.

## Introduction

**Fig 1:**
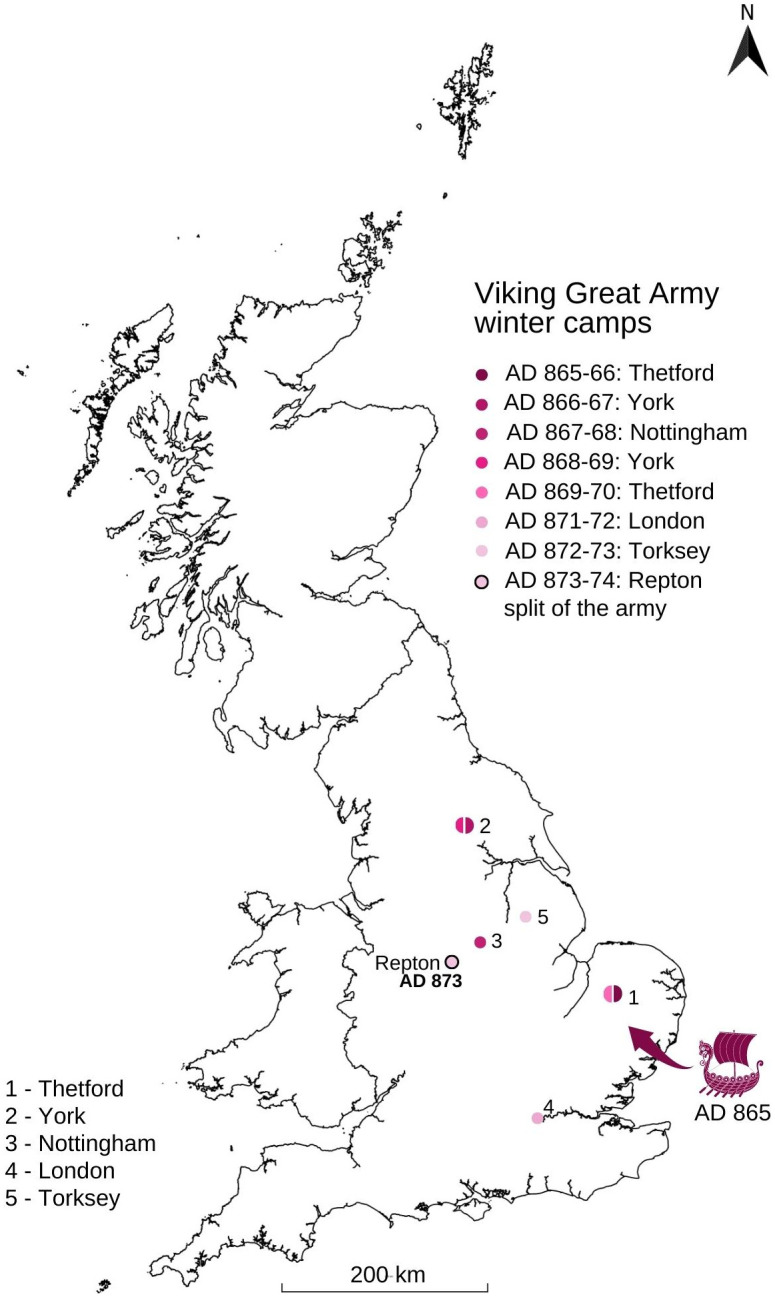
Location of Repton and the movements of the Viking Great army according to the Anglo-Saxon Chronicle.

The Anglo-Saxon Chronicle (hereafter ASC), our primary contemporary source, records that in AD 865 a Viking Great Army landed in East Anglia (Fig 1) [1]. Previously, raids had followed a pattern of hit-and-run, attacking vulnerable coastal monasteries, but this new army remained longer, overwintering at camps set within the heart of England. Over the following decade this force moved rapidly, travelling by land and water, and fighting each of the Anglo-Saxon kingdoms in turn, its strategy changing from the quest for portable wealth to the seizure of land for permanent settlement. In AD 873, the ASC records that ‘In this year the army went from Lindsey [in modern Lincolnshire] to Repton and took up winter quarters there’ [1]. An earthwork-enclosed area containing Scandinavian-style inhumation graves has been excavated at Repton by Biddle and Kjølbye-Biddle [2]. To the west of the enclosure lay an early medieval mausoleum which contained the disarticulated charnel of at least 264 adult individuals [3,4] (see SI.4 for more detail). Recent metal-detected finds, including characteristic lead gaming pieces and Islamic coins, as well as weights, now suggest that the area known as Foremark, situated between the village of Repton and the cremation cemetery at Heath Wood, was also part of the landscape that made up the camp at Repton [5]. In this historical and spatial context, however, it is Heath Wood which stands out through its unparalleled nature in the entirety of the British Isles. It is a unique Scandinavian cremation cemetery which features 59 mounds, and dates to a period by which Christianity had been firmly established in England, and inhumation was the exclusive burial rite–the ninth and early tenth centuries (Table 1). Heath Wood fundamentally contrasts with the generally more archaeologically invisible presence of Scandinavians in England, a matter of continuing interest in academic research [6].

**Table 1:** Radiocarbon dates for the cremated remains from mounds 11, 50 and 56 at Heath Wood (Richards et al 2004) [7].

Mound	11	50	56
Material	cremated human bone	cremated human bone	cremated human bone
Lab code	OxA-12698	OxA-12700	OxA-12699
Result (Conventional Radiocarbon Age)	1247 ± 27 BP	1191 ± 26 BP	1163 ± 26 BP
Calibrated age (95.4%)	AD 680–880	AD 770–950	AD 770–980

**Fig 2:**
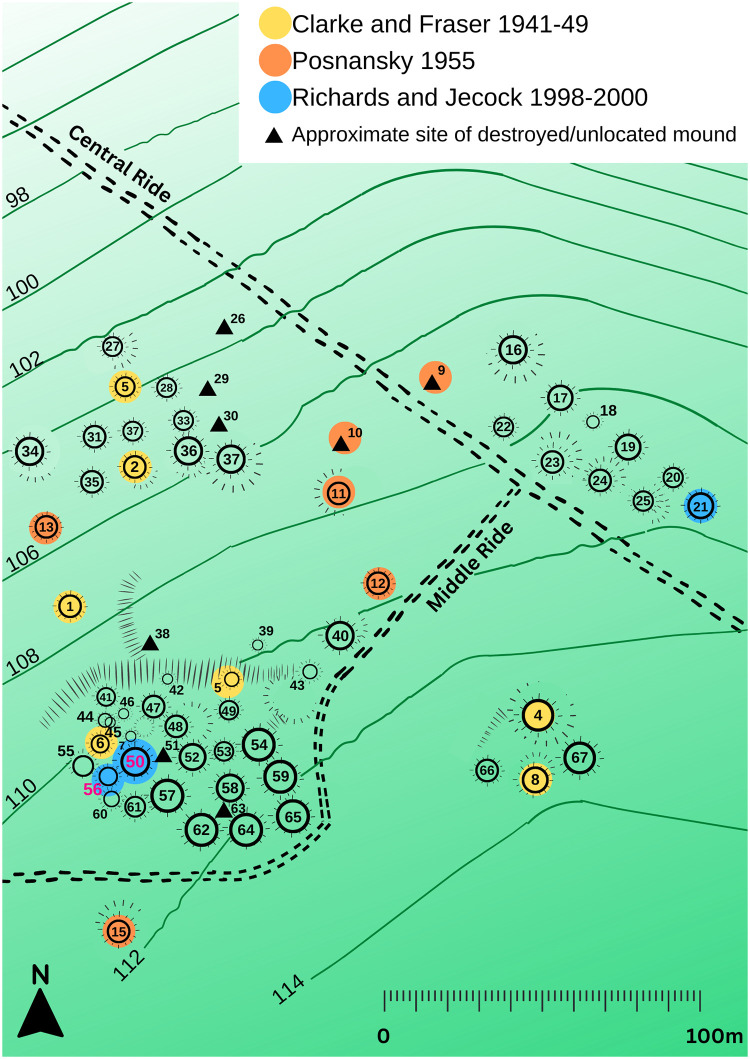
Plan of the Heath Wood site including all known mounds. Colour coding indicates excavated mounds during the different seasons (see also Fig 15 in [7]).

At Heath Wood, a total of 59 mounds are separated into four clusters (Fig 2) [7]. Only twenty of the mounds have so far been investigated, and most of this excavation took place in the 1940s and 50s [8,9] (see SI.5 for more detail). Calcined remains found within three of the mounds were radiocarbon dated to the eighth to tenth centuries AD (Table 1) [7]. All three dates are consistent with the Heath Wood cemetery being contemporaneous with the arrival and presence of the Viking Great Army at Repton in AD 873–4 as reported in the ASC. At the same time, the mound burials, cremation pyres, and animal remains accompanying the deceased are in stark contrast to the Viking inhumations around St Wystan’s shrine at nearby Repton where graves are oriented west-east and are located at the eastern end of the church. The individuals interred here are extended, supine and accompanied by Scandinavian grave goods–albeit without accompanying animals. Some of the individuals exhibit clear evidence of sharp force trauma (Grave 511) [2,3]. It has been suggested that the adoption of various mortuary rites may reflect the different warbands which made up the army [7]. Indeed, the ASC records that on its departure from Repton in the spring of AD 874, the army split in two.

The excavations revealed that the mounds fall into two or three categories; while some include layers of burnt material including soil, charcoal and bone indicative of *in situ* pyres, others were apparently empty or included only a small amount of cremated bone. The latter have been interpreted as token deposits, which represent a body cremated elsewhere.

**Fig 3:**
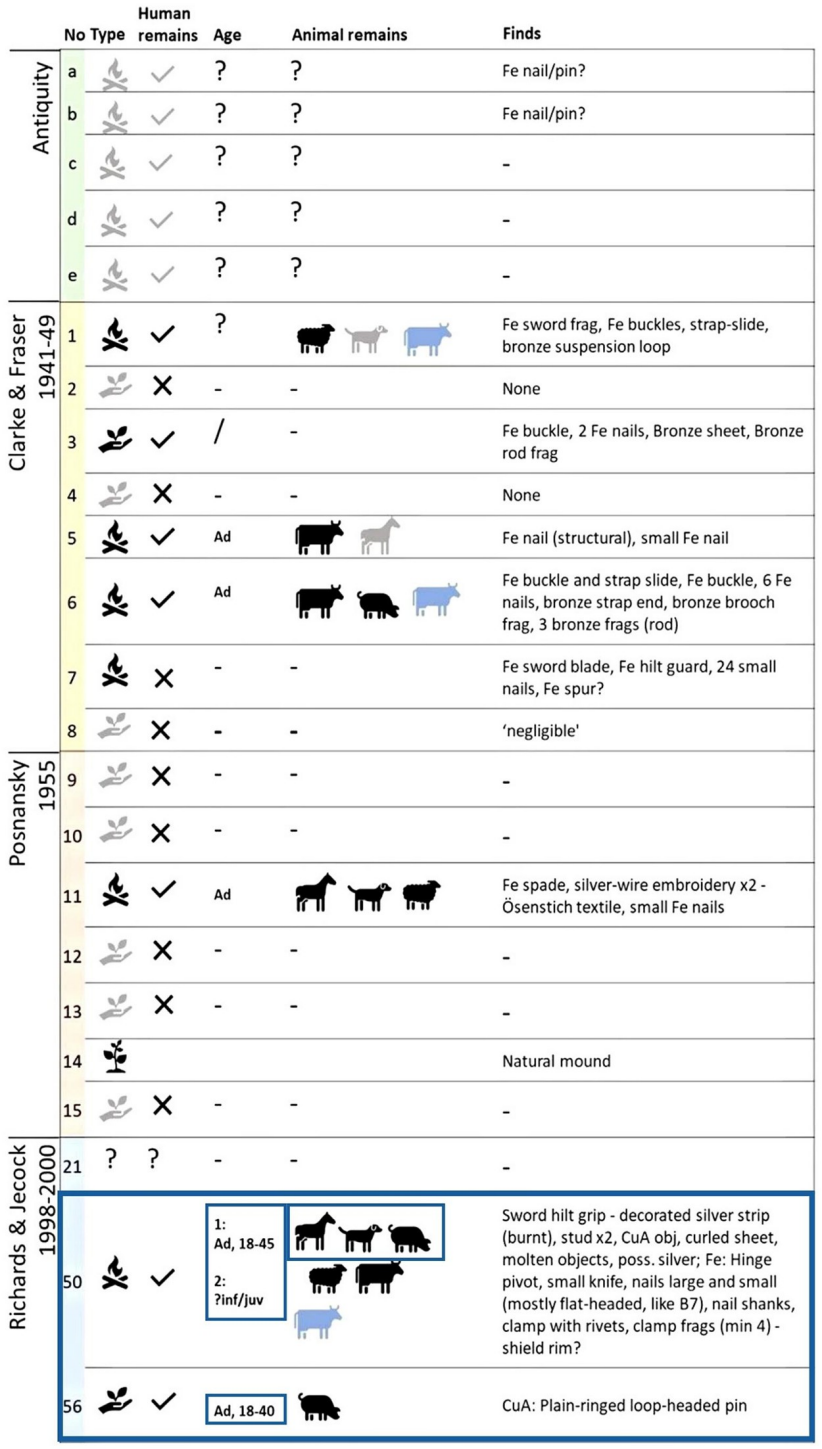
Summary of content of mounds at Heath Wood, colours of mound numbers/letters indicate excavation [7–9,13]; 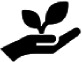
—Token burial; 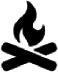
—Pyre in situ; 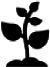
—Nat. mound; Grey symbols indicate a level of uncertainty. Light blue animal symbols indicate unburnt bone. Ad–Adult; inf–infant; juv–juvenile. Blue frame indicates sampled mounds and individuals.

During the excavations in the 1950s [9], only Mound 11 was noted to have an *in situ* cremation hearth at its centre, and subsequent analysis of the cremated remains by specialists McKinley, Bond, and Worley confirmed that these belonged to a human adult, a horse, a dog, and a sheep/goat [7]. A similar selection of animals and humans was recovered during the excavation of two further mounds– 50 and 56 –conducted by Richards and Jecock between 1998 and 2000 [7]. Not all the excavated mounds contained artefacts, but evidence for weaponry, with fragments of swords and shields, as well as dress accessories, nails, and other iron objects have all been recorded from the earlier excavations (Fig 3). Unfortunately, the only remaining cremated bone still available at Derby Museum derives from the latest phase of excavation. However limited this small sample of individuals may be–it holds the potential to illuminate our understanding of the site for the first time in decades, as a result of recent advances in isotopic analysis of cremated human remains [10–12].

The presence of calcined bone at Heath Wood allows the investigation of strontium (Sr) isotopes as tracer of residential origins of the cremated individuals. Sr is incorporated into mammalian tissues by replacing calcium (Ca) in the bioapatite lattice during (re)modelling of bone and the formation of teeth. It moves from the bedrock via food and water into the digestive system, whereby plants generally contribute the largest amount of Sr due to high dietary fibre and phytates content [14,15]. This is the case because Sr intake in the food chain is reduced with each trophic level through biopurification of Ca [16–18]–for example in a meat or dairy-rich diet [19]. The Sr isotope ratios (^87^Sr/^86^Sr) from the bones and teeth of an individual, who grows up while ingesting plants from the immediate surrounding area, should reflect the local biologically available Sr (hereafter BASr). By measuring these ratios in plants from the ‘local area’, it is possible to define the local BASr and its variations around the site [20,21]. In the past few decades, the use of ^87^Sr/^86^Sr on tooth enamel has become a well-established method to investigate childhood origins of inhumed individuals [14,22,23]. In 2015, Snoeck *et al*. [24] demonstrated that in contrast to unburnt bone, cremated bone represents a suitable substrate for strontium isotope analyses once an individual has been fully cremated (i.e. when the bone has turned white) due to the increased crystallinity of the bone matrix which even surpass that of tooth enamel. Therefore, ^87^Sr/^86^Sr and Sr content ([Sr]) measured in calcined bone reflect the *in vivo* accumulated Sr. In contrast to investigations of purely childhood/adolescence derived Sr (as in tooth enamel) the focus here lies on later periods of an individual’s life because of the modelling and remodelling processes of bone. In the following, we are tackling whether Sr isotopes can give us an indication where the individuals buried at Heath Wood came from.

## Materials and methods

### Human and animal remains

Only a limited amount of cremated bone was available for analysis. In Mound 56, McKinley identified an adult between 18 and 40 years [7]. A cross-section of the femoral shaft and a cranial fragment were sampled for this individual (see Table 2, Fig 3). In Mound 50, McKinley identified the remains of an adult between the ages of 18 and 45 for whom we sampled rib and femur. Additionally, a cranial fragment of a child who was identified by McKinley was used for Sr isotope analysis [7]. This individual was a probable ‘?infant/juvenile’ [7] and therefore likely younger than seven years old. Three of the animals found intermingled with the cremated human remains in Mound 50 were also included for ^87^Sr/^86^Sr analyses. The remains belonged to a horse, a dog, and a possible pig. The evidence from the excavation suggests that the horse, and most likely the pig (one half present), were cremated in full [7]. However, the same could not be confirmed for the dog because the pyre was raked and remains were partially removed post-cremation [7].

**Table 2:** Summary results for the cremated human and animal bone samples including age, location, bone element, ^87^Sr/^86^Sr and [Sr] data.

Name	Age	Mound	Bone element	^87^Sr/^86^Sr	2σ	[Sr] ppm*
HW00_242	Adult (18–40)	56	Femur	0.710314	0.000008	107.7
HW00 508 SF220			Cranium	0.710419	0.000009	156.0
HW99 411_1	Adult (18–45)	50	Rib	0.713655	0.000011	297.1
HW99411_2			Femur	0.714268	0.000010	260.0
HW99 411.2	?infant/juvenile	50	Occipital	0.710695	0.000009	115.0
HW99 411_2_P	-	50	Pig astragalus	0.716065	0.000008	272.0
HW99 411_2_H	> 3 y	50	Horse radius/ulna shaft left	0.715815	0.000010	287.2
HW99 308_D	> 11 m	50	Dog radius	0.715024	0.000007	256.0

*[Sr] normalised to 40% Ca by wt. to account for organic loss.

A sample of cortical bone from the fused radius/ulna fragment of the horse was used and based on the fusion stage, it was determined to have been older than three years [7]. The identification of the horse was re-confirmed by Bond for this study (*personal communication)*. For the dog, a fragment of cortical bone from the radius was used. The dog was older than 11 months at the time of death based on the stage of fusion of the distal radius [7]. A fragment of astragalus was analysed for the possible pig. Permissions for this study involving the destructive analysis of archaeological remains was sought from Durham University’s ethics committee (Department of Archaeology), and the study was conducted in accordance with relevant guidelines and regulation. The human and animal remains date to the ninth century AD and are currently under curation at Derby Museum. Upon consideration of our project proposal, the curators granted their permission for the destructive sampling of the remains. All necessary permits were obtained for the described study, which complied with all relevant regulations. Photographs of a selection of the cremated remains are available in SI.2 (Fig S2.1-S2.4 in S1 File).

### Plant samples

**Fig 4:**
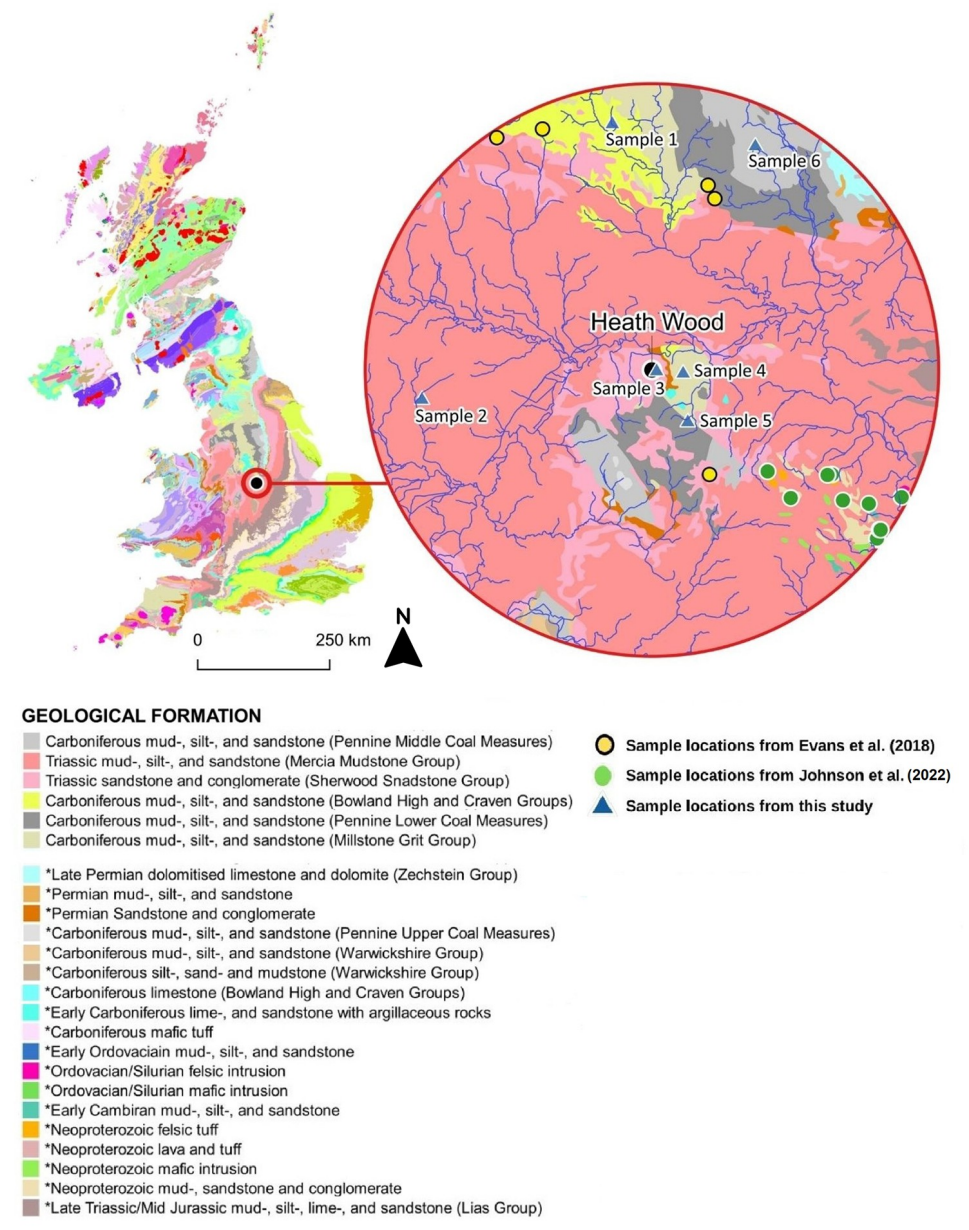
Bedrock map highlighting location of Heath Wood at its centre (black circle) and the 6 different plant sampling sites for this study covering the most extensive lithologies in the area (blue triangles). Extent denotes 25km catchment. Contains British Geological Survey materials (c) UKRI 2022.

To establish the variations within the local BASr, plants were collected from six locations within a 25km catchment area around Heath Wood (Fig 4) following the procedure described in Snoeck *et al*. [21]. Locations close to fertilised fields were avoided due to potential issues relating to Sr contamination through fertilisers, although it appeared later that location 6 was the site of 19^th^ and 20^th^ century mining operations, suggesting that the samples might not be suitable. Full details on the methodology can be found in the supplementary material (SI.1 and SI.2). Plant sampling was undertaken on public land in accordance with UK legislation under the Wildlife and Countryside Act (1981) which permits this, as samples consisted only of parts of plants–that is leaves and stems. None of the plants were uprooted and sampling did not take place in National Nature Reserves (NNRs) or Sites of Special Scientific Interest (SSSIs). Furthermore, none of the sampled plants were protected by Schedule 8 legislation (UK Wildlife and Countryside Act 1981).

### Methods

The calcined bone fragments were cleaned following the pre-treatment laid out in Snoeck et al. [10] using acetic acid and ultrasound at the Isotope Laboratory at Durham University. Subsequently, they were transported to the Analytical, Environmental and Geo-Chemistry (AMGC) department of the Vrije Universiteit Brussel (VUB) where they were individually powdered with a marble mortar and repackaged in 1.5ml plastic tubes. The powdered samples were then weighed out (ca. 15mg) for Sr content and isotope analyses. The weighed samples were placed in a Teflon beaker and digested in 1mL of subboiled 14M HNO_3_ and after dissolution, placed on a hotplate at 100°C until dryness. The plant sub-samples were homogenised by type to a total number of eighteen (three per site) in the laboratory facilities at the VUB in Brussels. An amount of 500mg was used for this process. The plant material was then transferred into Microwave cartridges and 4mL of subboiled 14M HNO_3_ were added. The microwave (Anton Paar Multiwave GO Plus) was run for 45 minutes after which the cartridges were removed and opened. 1mL subboiled 23M HF was added and placed in the microwave again for 45 minutes to complete the dissolution. The solution was then transferred back into the Teflon beakers and left to dry on a hotplate at 100°C. When the bone and plant samples were completely dry, strontium was extracted and purified following the protocol described in Snoeck et al. [10] and measured o n a Nu Plasma MC-ICP Mass Spectrometer (Nu015 from Nu Instruments, Wrexham, UK) at the Université Libre de Bruxelles (ULB).

Repeated measurements of the NBS987 standard were taken in the course of this study and yielded ^87^Sr/^86^Sr = 0.710246±0.000045 (2SD for >300 analyses). This result is sufficiently consistent with the mean value of 0.710252±0.000013 (2SD for 88 analyses) obtained by TIMS (Thermal Ionization Mass Spectrometry) instrumentation [25] for the purposes of this study. Each sample measurement was normalised using a standard bracketing method with the recommended value of ^87^Sr/^86^Sr = 0.710248 [25]. Procedural blanks were considered negligible (total Sr (V) of max 0.02 versus 7–8V for analyses, i.e. ≈ 0.3%). The ^87^Sr/^86^Sr value for each sample is reported with a 2σ error (absolute error value of the individual sample analysis–internal error). Fractions of the bone digested samples were used to determine Sr and Ca content in the sample digests with a Thermo Scientific Element 2 sector field ICP mass spectrometer at the Vrije Universiteit Brussel (VUB), Belgium, in low (^88^Sr) and medium (^42^Ca) resolution using Indium (In) as an internal standard and external calibration versus various reference materials (SRM1400, CCB01). The strontium content were calculated by normalizing the calcium data to 40% by weight to account for organic loss (see [26]). The analytical precision of the procedure outlined above is estimated through repeated digestion and measurement of two internal bioapatite standards (ENF and CBA) and does not exceed 5% (1SD, n = 33 for CBA and n = 5 for ENF).

### Mapping

For the maps we used QGIS software (version 3.10.4, ‘A Coruña’) and British Geological Survey (BGS) Geology 1:625 000 data which is available to download for Great Britain and Northern Ireland. All maps used for this study fall under UK Open Government Licence.

## Results

### Plants

**Fig 5:**
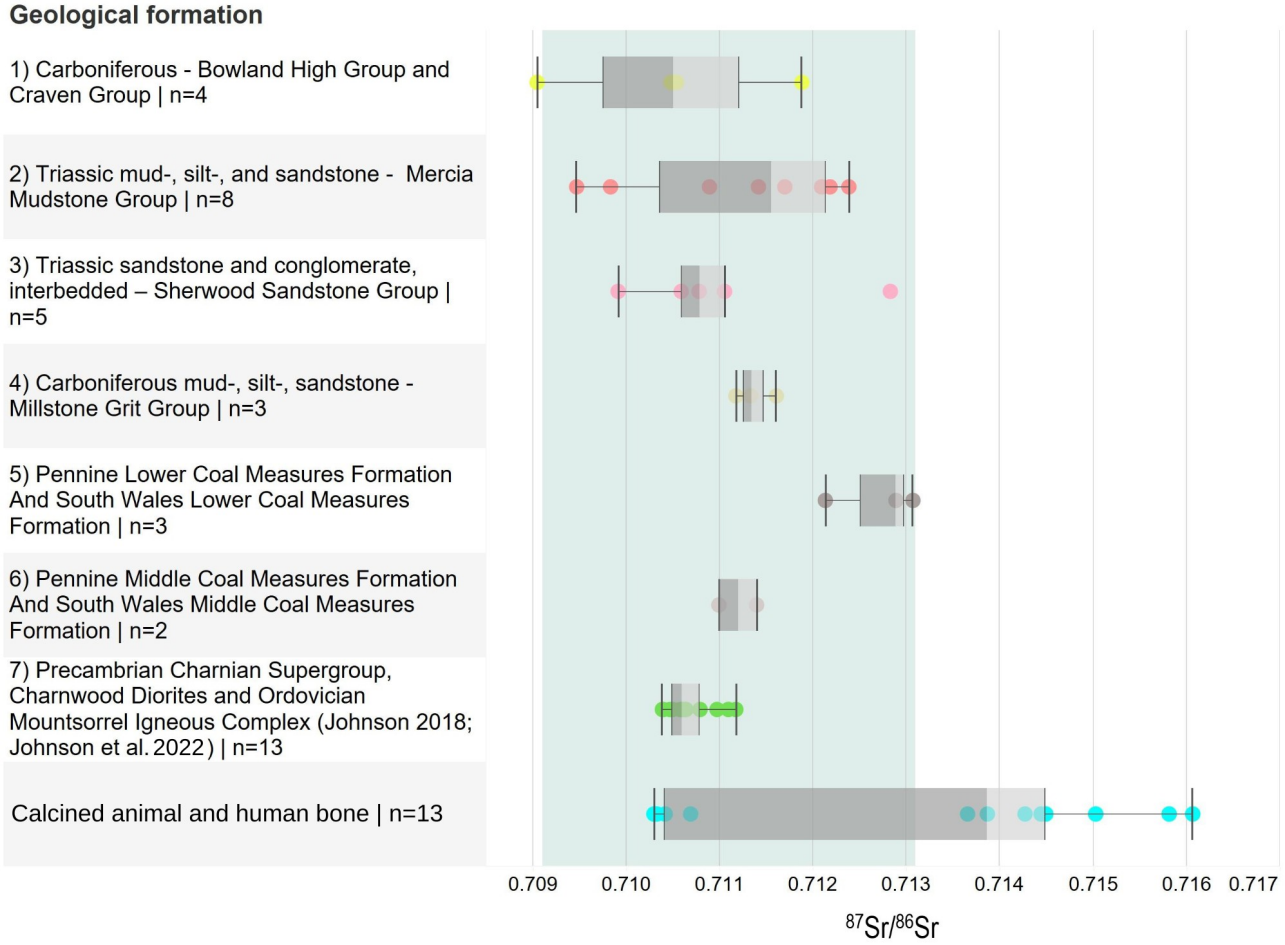
Boxplot of results from plant samples: ^87^Sr/^86^Sr by lithology including plant samples from BGS within or adjacent to the catchment area where available [27,31]. Location 6 samples from this study not included. Additional samples from Johnson [29,30] from the Charnwood area (see Fig 4). For information on lithology and sample location see Fig 4 and SI.3—S1 Table in S1 File.

The plant samples from the six different locations from within the 25km catchment of Heath Wood range from 0.7095 to 0.7151 (SI.3 –in S1 Table in S1 File; Fig 5). When previous plant data from Evans *et al*. [27,28] and Johnson [29,30] is included, the BASr ranges from 0.7091 to 0.7151. The highest ^87^Sr/^86^Sr value was derived from the grasses picked at sample location 6 on the Pennine lower coal measures, while the lowest ^87^Sr/^86^Sr came from a plant sample on the Carboniferous Bowland High Group and Craven Group [27,28].

The most extensive bedrock geology in the 25km catchment is the Triassic Mercia Mudstone Group (Sample 2; Fig 5; SI.3—S1 Table S1 File), which ranges between 0.7095 and 0.7109 based solely on our plant samples, and between 0.7095 and 0.7124 when including samples from Evans *et al*. [27,28] and Johnson [29,30]. Due to its proximity to Repton and Heath Wood, the results from sample location 3 are also highly relevant. These plant samples, based on the Triassic Sherwood Sandstone Group, range from 0.7106 to 0.7111 and fall between the other plant ^87^Sr/^86^Sr available on the same bedrock within the 25km catchment [28] (SI.3—S1 Table S1 File). The plant samples collected from location 6 (old mining site) on the Pennine Lower Coal Measures range from 0.7145 to 0.7151, and are higher in comparison to the plant ^87^Sr/^86^Sr based on the same geological bedrock, albeit c. 50km north of the site [28], with a difference of approximately 0.0030. They also contrast with data collected by Johnson [29,30] from the Pennine Coal Measures around Nuneaton, at 0.7103 and 0.7106, immediately south of the 25km catchment border. A further 12 plant samples based on the Precambrian Charnian Supergroup and the Charnwood Diorites southeast of Heath Wood range between 0.7104 and 0.7112, whilst a singular plant at 0.7110 is found on the Ordovician Mountsorrel Igneous Complex [29] (Fig 5; SI.3—S1 Table S1 File).

### Human and animal bone

**Fig 6:**
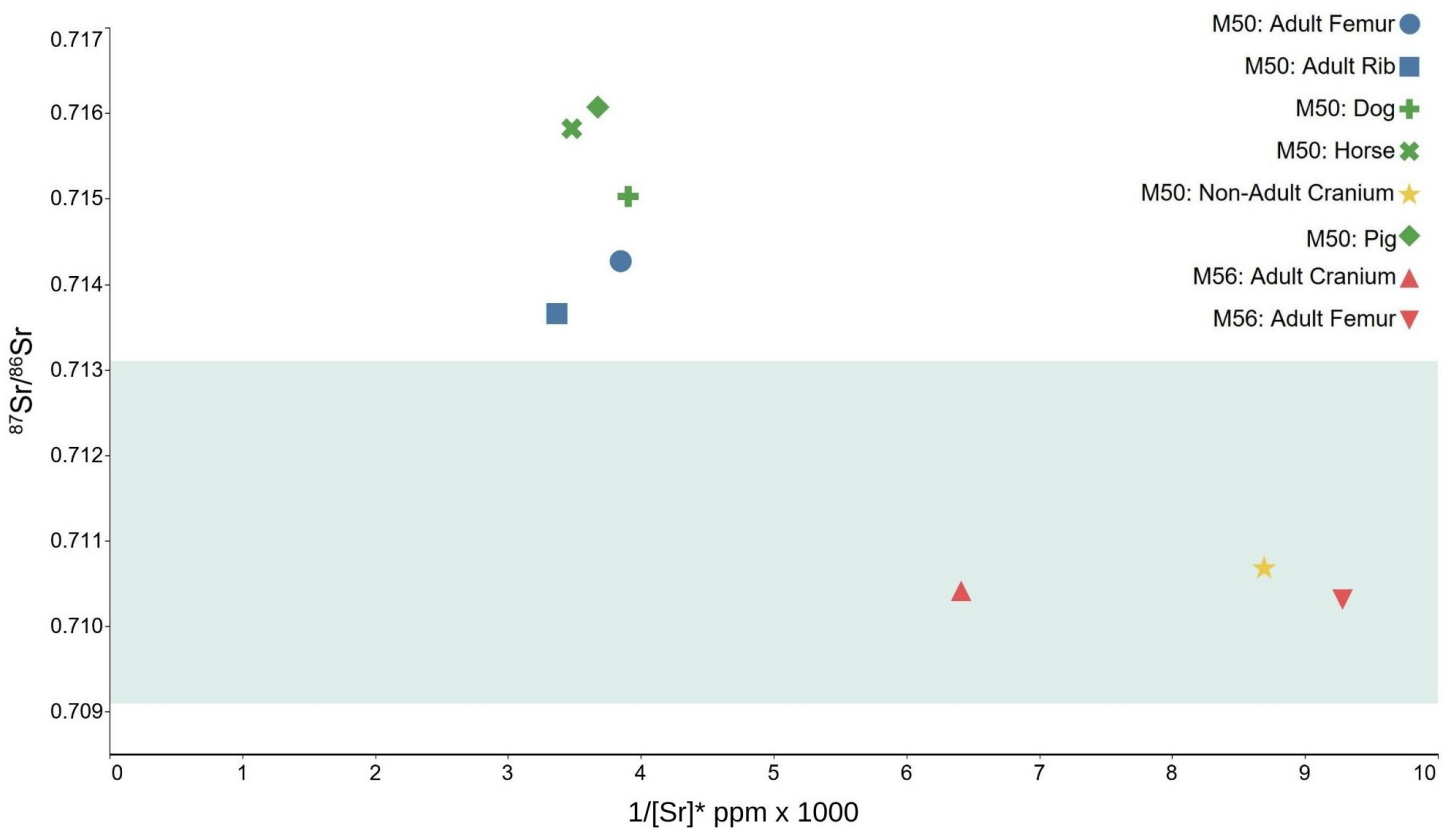
^87^Sr/^86^Sr vs [Sr] (expressed as 1/[Sr] x 1000) for the humans and animals of Heath Wood. Transparent green band indicates Sr biosphere within a 25km catchment of Heath Wood. M50 –Mound 50; M56: Mound 56. Error lies within the symbols. *[Sr] normalised to 40% Ca by wt. to account for organic loss.

The ^87^Sr/^86^Sr results measured on animal and human remains for Mounds 50 and 56 range from 0.7103 to 0.7161 (Table 2; Fig 6). The results for the adult individual in Mound 56 are lowest, with a ^87^Sr/^86^Sr of 0.7103–0.7104. The highest ratios are derived from the bone samples of the possible pig (0.7161), the horse (0.7158), and the dog (0.7150). The femoral ratios from the adult in Mound 50 are slightly higher than that of the rib sample from the same individual (Table 2). The cranial fragments from both mounds are similar in terms of their ^87^Sr/^86^Sr ratios (0.7107 and 0.7104 for Mounds 50 and 56 respectively). The Sr content (Table 2) further separate the individuals.

## Discussion

### BASr

An isotopic study by Budd *et al*. [32] included the oxygen and strontium (Sr) analysis of four individuals recovered from the charnel and four from the churchyard at Repton (raw data available in Evans *et al*. [33] and in Montgomery *et al*.: supplementary material [23]). The study included only a single soil leachate from Repton, which was considered representative of the local bioavailable strontium (hereafter BASr) with a ^87^Sr/^86^Sr value of 0.7115. More recently, Jarman *et al*. [34] proposed that the BASr for Repton ranged between ^87^Sr/^86^Sr 0.7112–0.7120, based on three dentine samples, one previously published soil leachate, and one faunal sample (cow). Since Budd *et al*. [32] was published, however, plant sampling has proven the best method to investigate local BASr because of its more direct representation of actually ingested Sr [35–38]. Our study therefore includes BASr estimation through plant sampling only.

The plant samples taken at location 6 returned unexpectedly high ^87^Sr/^86^Sr ratios, and clash with those measured by Evans *et al*. [27,28] for the same geological formation (SI.3—S1 Table S1 File). Further investigation into the site showed that sample location 6 served as a nineteenth century mining site. It is unlikely that the high values seen at location 6 are the average representative of the BASr on the Pennine Middle Coal Measures (discussion in supplementary material). Excluding the three samples from location 6, the variation seen in the plants from all locations measured in this study, including those measured by Evans *et al*. [27,28] and Johnson [29,30], ranges from 0.7091 to 0.7131 (Fig 5). We are here using this as a conservative BASr range. As such, any individual with ^87^Sr/^86^Sr lower than 0.7091 or higher than 0.7131 are deemed to not have resided long in the area around Heath Wood.

More generally, the underlying lithologies of Britain produce a biosphere ^87^Sr/^86^Sr range of 0.7070 to 0.7200 [27], but because of the nature of how resources and people are distributed in the landscape and the types of land available for settlement and agriculture, people and animals do not share this range as the higher values tend to come from upland regions of granites and gneisses. In 2012, Evans *et al*. [33] published a review of ^87^Sr/^86^Sr results deriving from individuals in Britain. The highest ^87^Sr/^86^Sr value from England and Wales was noted to be 0.7140 (Hereford Cathedral), with values from Scotland sometimes exceeding those of the rest of Britain. More recent studies have found that ^87^Sr/^86^Sr ratios exceeding 0.7165 are unlikely for British individuals [39,40]. Depending on how mobile individuals were in the past and how locally derived their food sources were, it would in theory be possible for people living and growing up in England to have a higher ^87^Sr/^86^Sr than 0.7140 –but this would presuppose that they remained stationary on a very small catchment all their lives, for example on the Devonian red sandstones of Herefordshire, Somerset, and Cornwall (located approximately 74.5km, 230km, and 300km away from Repton respectively). This would be very unlikely for members of the Viking Great Army, which is known to have been very mobile and/or composed of recent incomers [1,7].

### Sr isotopes of human and animal remains

The adult and the animals in Mound 50 have ^87^Sr/^86^Sr ranging from 0.7137 to 0.7161 and are conclusively not from the area around Heath Wood. The nearest areas to Heath Wood that consistently produced BASr values >0.7130 include the Precambrian bedrock of the Malvern Hills (England), the Silurian Ludlow bedrock in Wales [27,29,41], the Carboniferous granitic intrusions of Dartmoor [42] or further afield towards Scotland [27,29] and Ireland [21]. However, when viewed in consideration of their archaeological context, and for reasons pertaining to how ^87^Sr/^86^Sr are generated in tissues from the environment, it is likely that these ratios were not produced in the British Isles. Instead, the ^87^Sr/^86^Sr observed most likely reflect a life on the Palaeozoic and Precambrian gneissic or granitic lithologies of Scandinavia–an area with convincing parallels to Heath Wood in terms of burial rites and grave goods. These lithologies are common within the Baltic Shield (Palaeozoic and Precambrian lithologies), which underlies most of Norway and large areas of central and northern Sweden, and indeed Finland. BASr values from Scandinavia regularly produce values from 0.7140 to >0.7200 [43–50]. It is thus convincing that the adult human and the animals buried at Heath Wood in Mound 50 came to Britain from this part of the world (Fig 6). In fact, the horse has a ^87^Sr/^86^Sr of 0.7158 and this may be explained by a diet exclusively ingested by grazing on meadows on granitic or gneissic soils/lithologies. Similarly, the ^87^Sr/^86^Sr of the possible pig suggests a diet deriving from a high ^87^Sr/^86^Sr lithology. In early medieval rural locations, pigs have been noted to feed on acorns in forests, as well as on roots of ferns and dandelions [51], which can explain these values considering the observation that ancient woodland areas often produce elevated ^87^Sr/^86^Sr [29 Ch 6; 30]. The third animal, the dog, has a ^87^Sr/^86^Sr of 0.7150, slightly lower than that of the horse and pig but more elevated than the adult human, and this may be due the fact that dogs, like humans, are omnivores and feed off scraps from human food sources, especially if they are companion animals.

An alternative explanation pertaining to these values is that the animals could have died soon after arrival in Britain, without extensive bone remodelling taking place in the new environment. This would have made it impossible for their tissues to reflect the lower ^87^Sr/^86^Sr ratios of the local area. Different bone turnover rates in the specific bones sampled for the animals, or indeed death at different points in time before cremation may also play a role in determining the different ^87^Sr/^86^Sr ratios measured. By extension, through the process of bone turnover, ^87^Sr/^86^Sr from the catchment of Heath Wood could have contributed to produce the lower ^87^Sr/^86^Sr ratios seen in the adult. Bone remodelling is a relatively slow process, however, and therefore it is most likely that the difference in ^87^Sr/^86^Sr ratios was produced by a dissimilar diet, for example through a more substantial consumption of marine resources such as seaweed or salt with a ^87^Sr/^86^Sr of 0.7092 [14, 52]. There is also a slight decrease in ^87^Sr/^86^Sr from 0.7143 (femur) to 0.7137 (rib). It may be that this reflects mobility and/or a change in diet. If the ^87^Sr/^86^Sr are considered as a process and not as a ‘snapshot’, an explanation may be proposed: In the rib–reflecting the Sr intake of the last years of life (see SI.6)—a shift in diet, to crops with lower ^87^Sr/^86^Sr, can be recognised. The contribution of Sr from the new biosphere, or indeed from the diet during extensive travel, may have pulled the average of the ^87^Sr/^86^Sr towards a lower value, while the thick femoral cortex of the femur is subject to much slower turnover [53,54] and therefore reflects more substantially the elevated ^87^Sr/^86^Sr of the previous environment.

The ^87^Sr/^86^Sr of the adult (56) and the child (50) fall within the BASr range of the 25km catchment around Heath Wood, and more generally, are consistent with values seen across Britain [27,28]. It is conceivable that these individuals grew up in the area, in southern or eastern England—or even large parts of Europe. For Scandinavia, Denmark or southwestern Sweden would also be a possible place of origin as they share similar lithology and have a BASr range of 0.7081–0.7111 [55,56]. For the cremation rite observed at Heath Wood, parallels have been identified at the contemporaneous cemeteries of Northern Jutland, in Denmark [7,57], although mound cremations are also known from central Sweden and Norway. Crucially, this is a single-proxy study and unfortunately, it is impossible to be more precise about the origin of these two individuals. In terms of both ^87^Sr/^86^Sr and [Sr], the non-adult from Mound 50 is far removed from the adult and the animals found in the same mound that have much higher ^87^Sr/^86^Sr (0.7137–0.7161). The ^87^Sr/^86^Sr in fact deny direct immigration from Denmark for the adult individual and animals in Mound 50, based on existing Sr biosphere data [55,56]. This is a strong indication that the two people in Mound 50 spent most of their lives in different places. The [Sr], which is tied to diet and soil/lithological composition strengthens this point: All three animals and the adult from mound 50 exhibit similar values. This is unusual, because most often a trophic level shift between humans and animals is observed, which is due to different proportions of protein and plant fibre content in their diets [19]. Additionally, the calcium content and weathering of soils and lithologies influence the uptake of Sr: while Ca-rich soils can limit the [Sr] in individuals, Ca-poor soils can have the opposite effect. Another variable to consider in this context is the weathering of different lithologies. As such, the adult from mound 50 may either have lived on and/or derived their diet from a low-calcium, relatively high ^87^Sr/^86^Sr lithology or eaten a diet rich in plants from a lithology with slightly lower ^87^Sr/^86^Sr than the animals. Depending on topography and the available land for grazing animals and growing plants for consumption, it is certainly possible that this was the case. In contrast, the [Sr] observable in the non-adult from mound 50 and the adult from 56 is much lower and shows that these individuals had a differently composed diet than all other individuals; coupled with the ^87^Sr/^86^Sr, it emphasises the procurement of food sources from a different lithology altogether.

Fundamentally, the Sr data presented here suggests that there are people and animals with different mobility histories buried at Heath Wood, and that—should they have belonged to the Viking Great Army—this war band was composed of people from different parts of Scandinavia and/or the British Isles. Recent work by Jarman *et al*. [4,33] has highlighted the same differential composition of the Scandinavian presence at nearby Repton. In contemporary Scandinavia and the Baltic, similarly varied mortuary populations have been detected at Birka [49], Sigtuna [58], at the early medieval trading port at Ridanäs, Gotland [59], at the Trelleborg fortress in Denmark [60], and in Salme in Estonia [61,62], amongst many others. In the British Isles there have been similar observations for this period at Weymouth [63], in Dublin [23] (although see Knudson [64]), Orkney, and the Hebrides [65]. Therefore, it is unsurprising to find people of different provenances across Europe during the Viking age. However, this research presents the very first direct evidence that not only people made their way across the North Sea in the ninth century, but also animals. This is of particular interest given the specific association between Heath Wood and the Viking Great Army of AD 865–78, and by extension the information contained in the ASC. As noted above, the Viking Great Army was a highly mobile force, travelling by a combination of land and water, and camping at strategic crossing points on arterial rivers, where mounted warriors could be resupplied from the slower moving fleet [66,67]. The ASC records that the force that first landed in East Anglia in AD 865 ‘took up winter quarters’ and ‘there they were supplied with horses’ [1]. Indeed, given the difficulty of transporting horses across the North Sea in open boats one might assume that the army generally seized its horses in England. However, it is not impossible that its leaders brought their personal mounts with them. A few decades later, in the entry for the year AD 892, the ASC reports that a part of the army moved from France to Kent on ships ‘in a single journey, horses and all’ [68]. Almost two centuries later, in vessels which still closely resembled Viking plank-built ships, the Norse descendent William of Normandy reportedly transported some 10,000 men and 2000 to 3000 horses across the English Channel, as famously depicted on the Bayeux tapestry [69]. In 1967, a horse was successfully disembarked from a replica of the Ladby ship [70], although it is unlikely that vessels with such a low freeboard could be used to transport horses in anything but the smoothest of conditions. The best-preserved examples of Viking ships with a hold and deep enough draught to transport livestock are the vessels known as Hedeby 3 (constructed c. 1025), Skuldelev 1 and 3 (both built c. 1040), Roskilde 3 (built after 1060) and Roskilde 4 (built 1108). However, the Norwegian migrants who settled Iceland from the 870s onwards took their cattle with them, and it is probable that the large fleets, which landed in England in the same period, included cargo ships as well as the seek and slender long ships. Indeed, archaeological evidence from excavated early Scandinavian ships indicates that amongst the ninth and early tenth century models, it was the Norwegian and Swedish examples, which appear to have had more significant loading capacity through more stabilising proportions [71–73]. Given that, it is less surprising that a Viking leader would also bring their prized hunting dog, another key status symbol. A similar instance was reported at Salme in Estonia by Price *et al*. [61,62]. Despite the less secure identification of the pig, it is reasonable to assume that the Army would bring food supplies with it, rather than depending entirely upon what it could pillage or trade for. The discovery of a hoard of three plough shares at the camp at Torksey, and a fourth at Foremark, might also indicate a level of intentionality to settle [66,74], and the pig might have been intended as initial livestock. Alternatively, it may have arrived as preserved food source or as amulet.

At Heath Wood, we observe a rite strongly symbolic in character. A selection process is implicit in the sacrifice of the animals in the mounds and, short of dying on the battlefield, their deaths do not make economic sense. This is the case especially in a scenario where the Viking Great Army–a group of people intent on settling in a new environment–lands in England with what was presumably a limited stock of animals in tow. By extension, a prior selective process is apparent which is based on the fact that a ship would only be able to carry a very limited number of these animals across the North Sea. Through the burial rite, and the inclusion of ‘companions’ from Scandinavia, the mounds at Heath Wood provide a direct link, a proxy, to the ‘homelands’ of those buried here [75–77]. This becomes especially momentous in the context of the inhumation burials of several individuals of Scandinavian origin–likely also members of the Viking Great Army–at Repton [4,5]. If the cemeteries are contemporaneous and were indeed created in the span of a few years, the conscious decision was made at Heath Wood to cremate rather than to inhume the dead, to create mounds above the burials, and to do so far away from the commemoration place of the Mercian royal line and St. Wystan in Repton. The use of outright Scandinavian burial practices and artefacts may indicate a looking backwards, or a reference to the old homelands [78]. The formation of what Ó Ríagáin terms ‘emotive foci’ in a contested landscape [78], through the deposition of the dead, creates a connection of people to a particular space.

## Conclusion

The cremation cemetery at Heath Wood presents a unique and robust fragment of evidence of the Scandinavian presence in early medieval Britain. In stark contrast with the omnipresent contemporary inhumation rite in Britain, the cremated bone inside the mounds indicates a strong Scandinavian influence. The ^87^Sr/^86^Sr analysis of the small amount of cremated bone has shown that the adult from Mound 56, and the child from Mound 50 had ^87^Sr/^86^Sr consistent with the local Sr biosphere and thus conceivably grew up and lived locally (although similar values are seen in Denmark and southwestern Sweden). However, in the context of the archaeology, the ^87^Sr/^86^Sr values of the other adult, the horse, the dog, and the possible pig in Mound 50 indicate migration from the Baltic shield regions of Scandinavia—that is Norway, or central and northern Sweden. This assessment is strongly supported by the contextual information of the site, such as the striking parallels of the funerary ritual of cremation in conjunction with mound construction, as well as the presence of Scandinavian style grave/pyre goods and historical documentation in the *Anglo-Saxon Chronicle*. Consideration of continuous bone remodelling throughout life suggests that these incoming individuals died soon after arrival. These results provide the first and unique evidence for the migration in the late ninth century of both people and their animals–including horses and dogs–across the North Sea, from Scandinavia to the heart of England.

## Supporting information

S1 Fig Photograph of femoral diaphysis fragment from Mound 50. (TIF)

S2 Fig Photograph of rib fragment from Mound 50. (TIF)

S3 Fig Photograph of horse radius/ulna fragment from Mound 50. (TIF)

S4 Fig Photograph of femoral diaphysis sample from Mound 56. (TIF)

S1 File (DOCX)
