# Supplementary figures and images for: Sr analyses from only known Scandinavian cremation cemetery in Britain illuminate early Viking journey with horse and dog across the North Sea
Source: PLoS One. 2023 Feb 1;18(2):e0280589. doi: 10.1371/journal.pone.0280589 (PMC9891522; doi:10.1371/journal.pone.0280589)

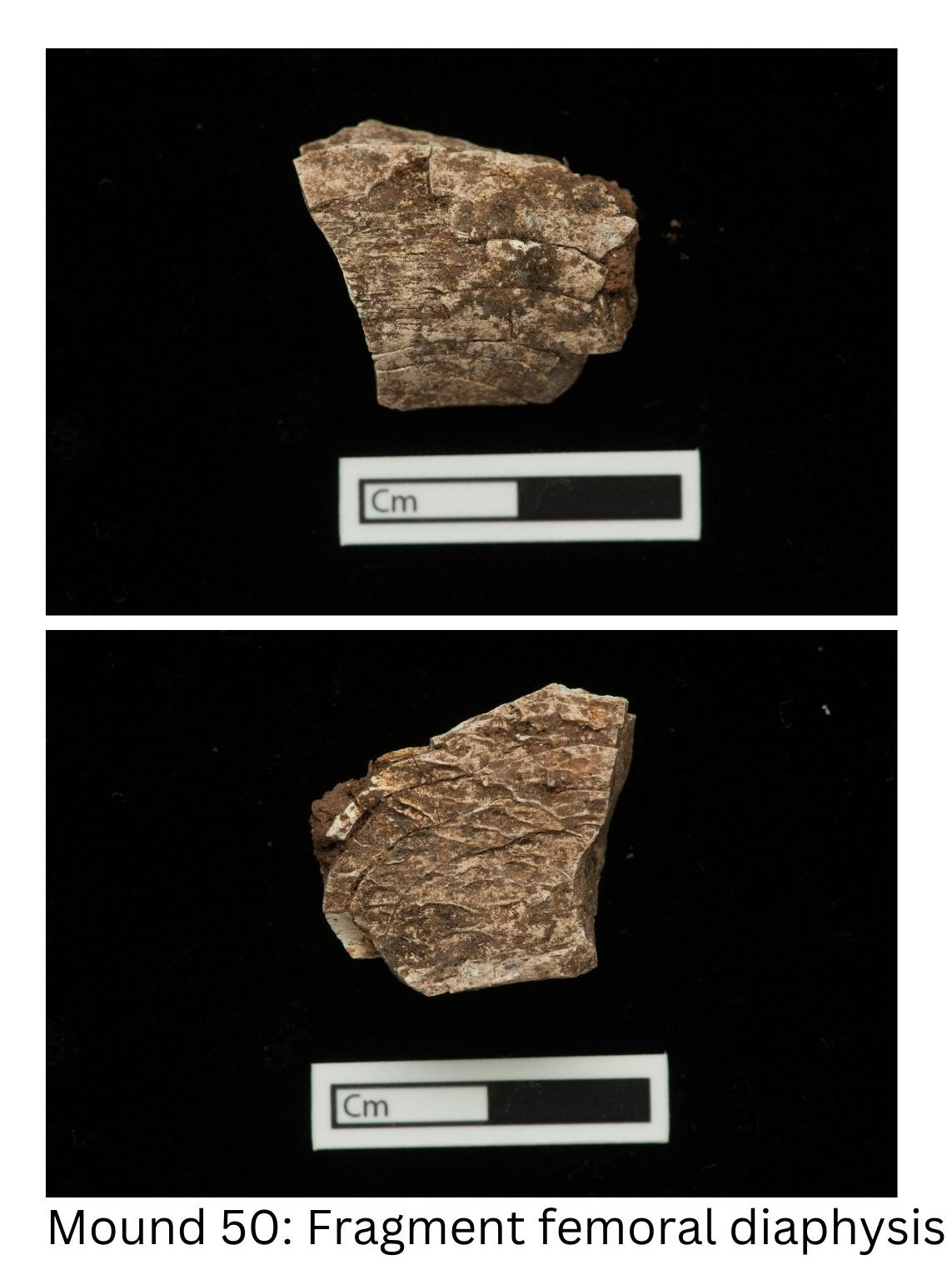

Supplement: S1 Fig — (TIF) [file pone.0280589.s001.tif]

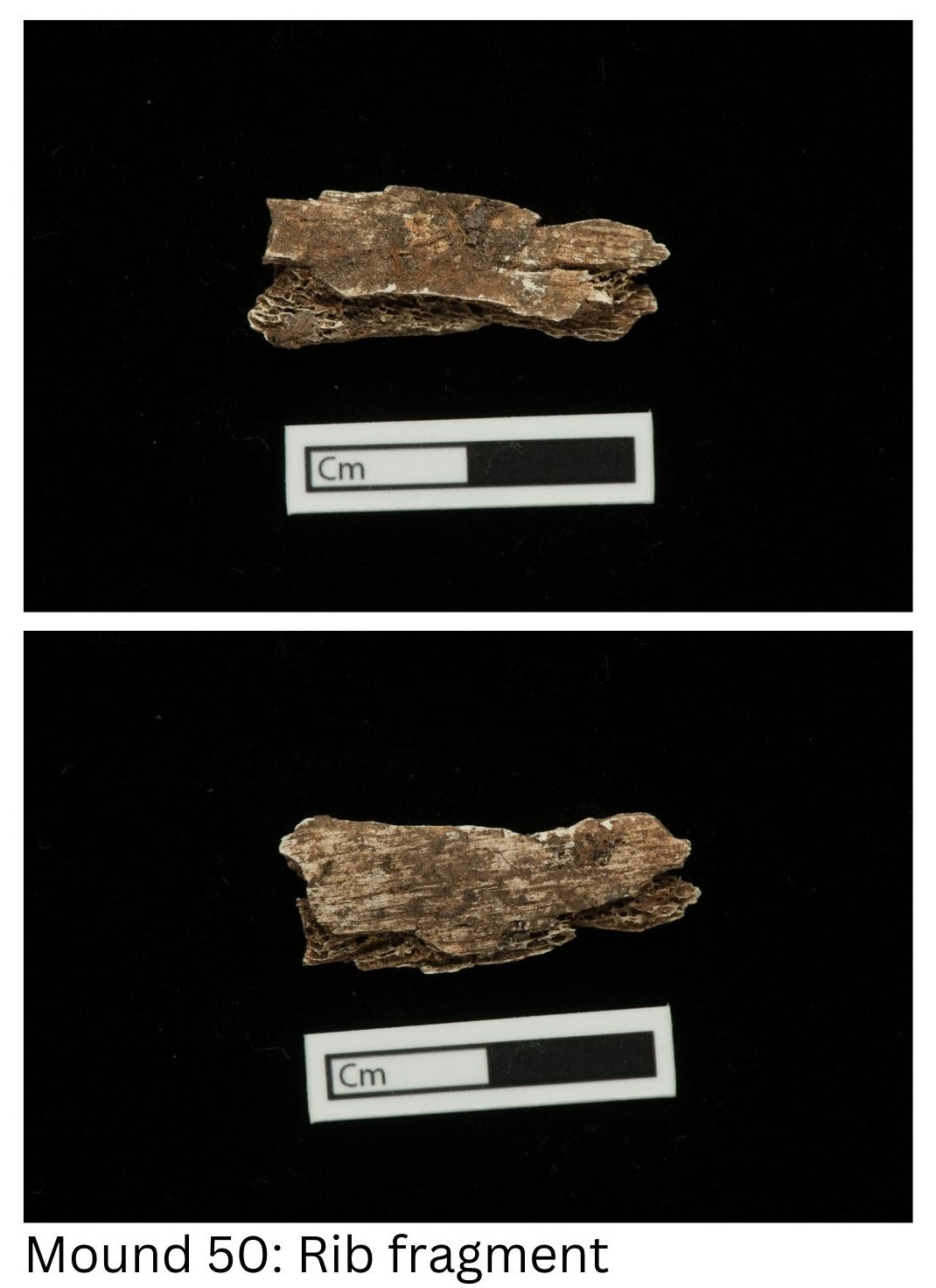

Supplement: S2 Fig — (TIF) [file pone.0280589.s002.tif]

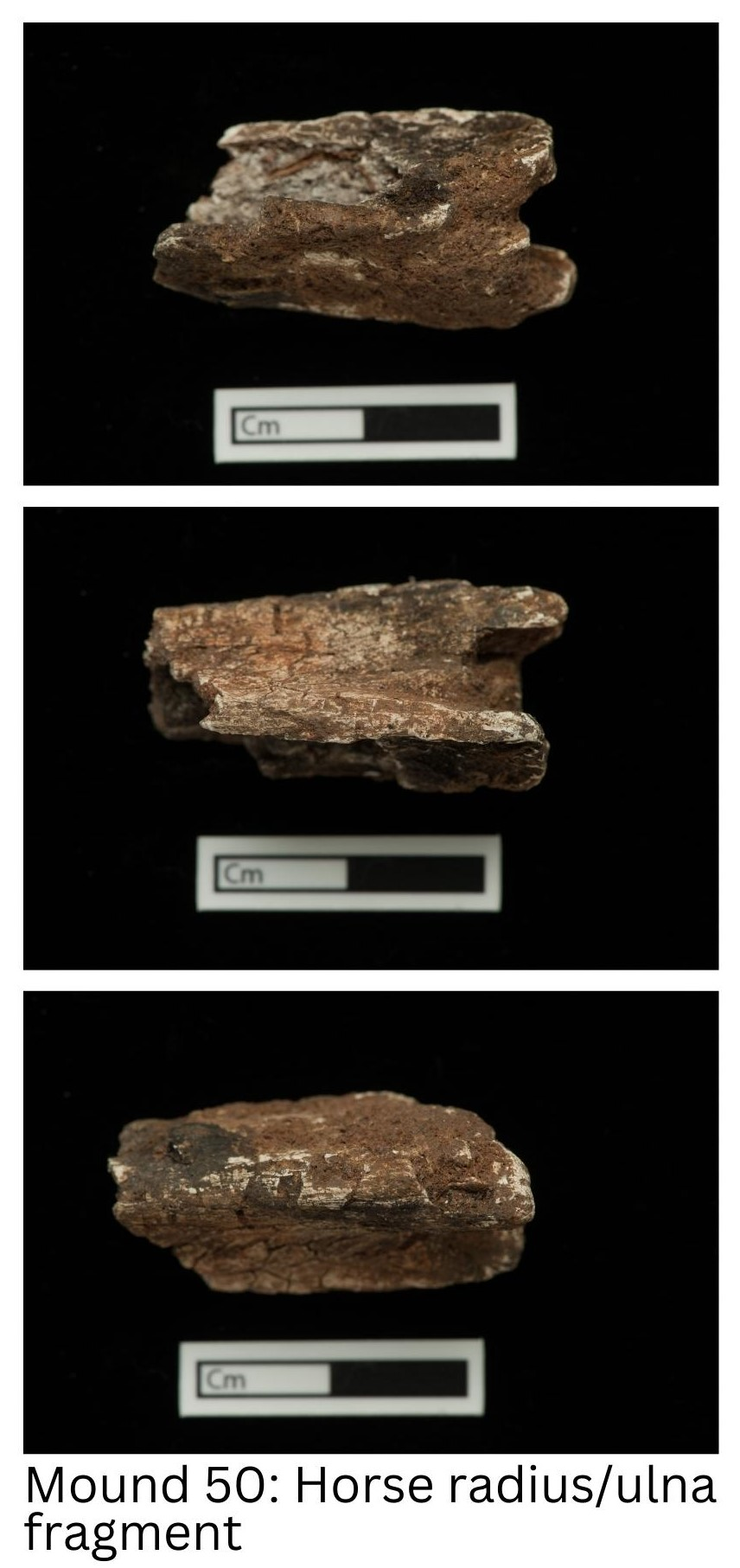

Supplement: S3 Fig — (TIF) [file pone.0280589.s003.tif]

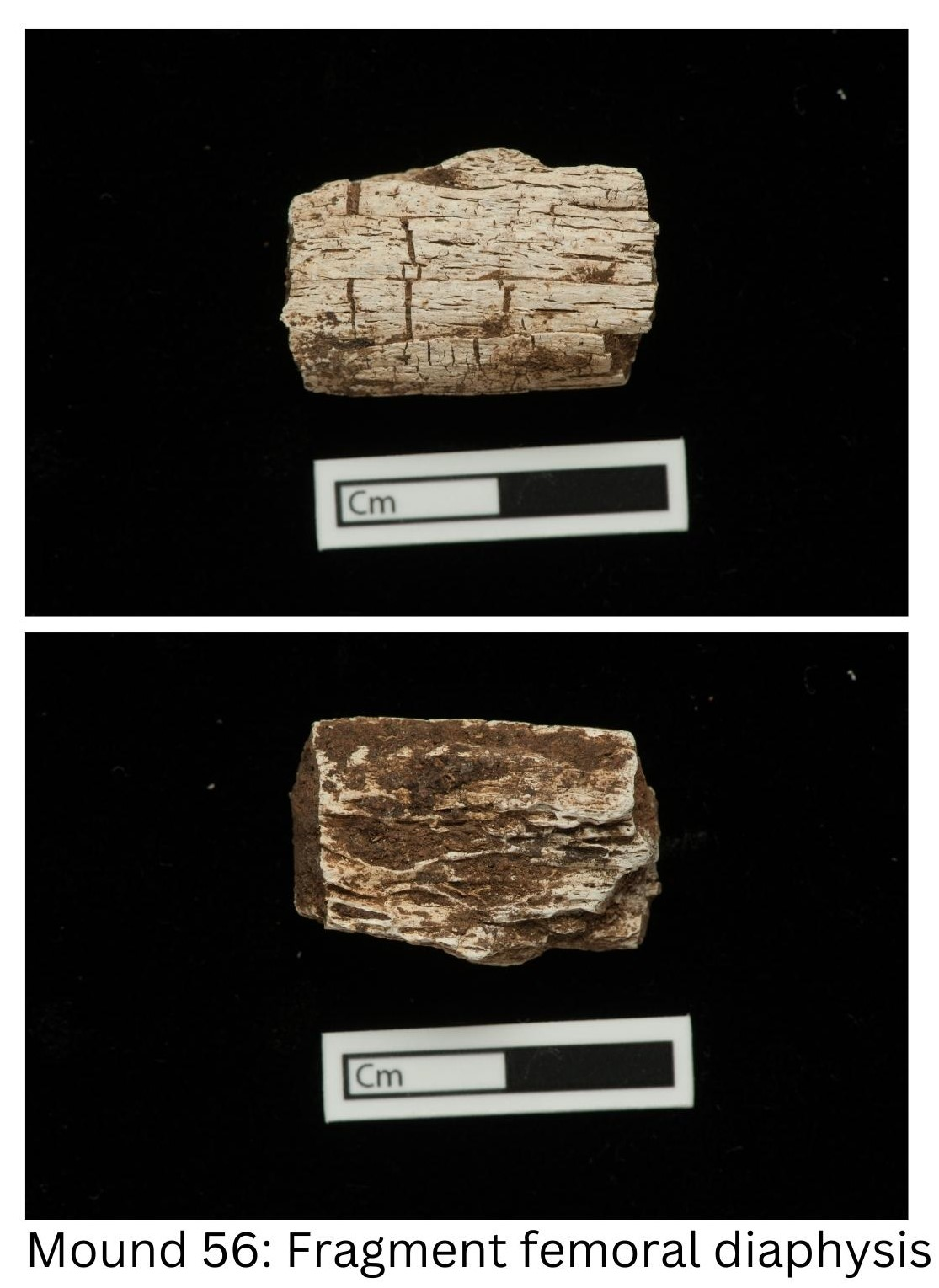

Supplement: S4 Fig — (TIF) [file pone.0280589.s004.tif]
