# Supplementary material for: Sr analyses from only known Scandinavian cremation cemetery in Britain illuminate early Viking journey with horse and dog across the North Sea
Source: PLoS One. 2023 Feb 1;18(2):e0280589. doi: 10.1371/journal.pone.0280589 (PMC9891522; doi:10.1371/journal.pone.0280589)
Supplement: S1 File — (DOCX) [file pone.0280589.s005.docx]

# **Supporting Information**

**SI.1) Method: Plant sampling**

For each sample location leaves were picked off three trees and three shrubs of the same species, as well as blades from three patches of grass. All these plants used were found within a radius of 50m of each other. The sample sites were pre-selected using Google Earth satellite imagery and BGS 625k Bedrock and Superficial Geology KML layers [1, 2]. To establish the variations within the local BASr, plants were collected from six locations within a 25km catchment area around Heath Wood (Fig. 4) following the procedure described in Snoeck *et al.[3]*. Locations close to fertilised fields were avoided due to potential issues relating to Sr contamination through fertilisers. Plant sampling was undertaken in accordance with UK legislation under the Wildlife and Countryside Act (1981), as samples consisted only of parts of plants – that is leaves and stems. None of the plants were uprooted and sampling did not take place in National Nature Reserves (NNRs) or Sites of Special Scientific Interest (SSSIs). Furthermore, none of the sampled plants were protected by Schedule 8 legislation (UK Wildlife and Countryside Act 1981), and no commercial gain was made from them. Identification of the plant species was confirmed by Dr Charlotte O’Brien (Durham University: *personal communication*).

## *Plant results Sample site 6:*

The plant samples taken at location 6 returned unexpectedly high ^87^Sr/^86^Sr ranging from 0.7145 to 0.7151. These contrast with the two plant samples collected by Evans *et al.* [1, 2] mentioned in the results (main text), and also contrast with data collected by Johnson [4] from the Pennine Coal Measures around Nuneaton, at 0.7103 and 0.7106, immediately south of the 25km catchment border. According to Evans et al (2018), carboniferous sandstones in central and north England produce ^87^Sr/^86^Sr ratios in plants which fall in-between 0.7095 (minimum value measured) and 0.7117 (maximum value measured; n=11). The carboniferous sandstones of SW England and Wales produce a range of 0.7103 to 0.7126 (n=7), and those of Scotland fall in-between 0.7091 and 0.7124 (n=17). Therefore, there exists significant doubt that the values measured at plant sample location 6 are a genuine representation of the underlying rock unit and by extension, the BASr.

All the sample 6 plants were collected in what today is the Shipley Country Park in Derbyshire. This is a communal park area with parking, a small expanse of grass lined with trees, and shrubs. Beyond a narrow strip of trees to the north-northeast lies a modern housing development. In the late 1800s, this was farmland around Thorpehill farm. By the late 1880s, Great Northern Railways had constructed the Heanor railway branch, which was not only used to transport passengers, but also coal and other materials between 1890 and 1925, after which it was only used sporadically and then fell into disrepair. When the tree, shrub and grass sample locations are superimposed onto historic maps, it becomes clear that the samples were taken from the exact run of the historic track of the Great Northern Railway (Heanor branch; Derbyshire HER: MDR4963; Monument record available at https://her.derbyshire.gov.uk/Monument/MDR4963). Interestingly, the lowest ^87^Sr/^86^Sr derived from the shrubs, which were located furthest from the railway track.

One attempt at an explanation related to the railway track directly concerns the construction materials used. Railway tracks are not placed directly on the ground, but rather upon a bank of built-up earth, and material which is known as ‘ballast’. Historically, different types of ballast were used, and primary importance was given to the size rather than the material. The different types of ballast included stone, broken brick, slag, ashes, cinder, ashes, sand, or clay, and sometimes a two-layered system was employed which meant that finer material was laid out atop the coarser material to stabilise the sleepers and facilitate load distribution [5]. There is a possibility that granite from northern quarries was used, although the transportation requirements make this an unlikely explanation. It is also possible that the material used to backfill the rail track after decommissioning in the 1960s is responsible for the high ^87^Sr/^86^Sr values. In conclusion, the location for our plant sample 6 is clearly heavily impacted on by anthropogenic processes and is in close proximity to longstanding industrial activity.

**SI.2) Photographs of selection of cremated bone samples**

**Figure S2.1 – Photograph of femoral diaphysis fragment from Mound 50.**

**Figure S2.2 – Photograph of rib fragment from Mound 50.**

**Figure S2.3 – Photograph of horse radius/ulna fragment from Mound 50.**

**Figure S2.4 – Photograph of femoral diaphysis sample from Mound 56.**

**SI.3) Table of plant samples**

**Table S1 - ^87^Sr/^86^Sr for the seven plant sampling locations.** For locations see map in Fig. 4. Plant samples from Evans et al[1, 2] located within the 25km catchment area are included for comparison with the new data. (*) Denotes samples located c. 50km north of Heath Wood from Evans et al. 2010. Sample location 7 data from [4].

| **Plant sample location** |  | **Coordinates** | |  | **Bedrock** |  |  | **^87^Sr/^86^Sr from plant samples (Evans et al 2010, 2018; Johnson, 2018)** |  | **^87^Sr/^86^Sr from samples from this study and 2SD** | | |
| --- | --- | --- | --- | --- | --- | --- | --- | --- | --- | --- | --- | --- |
|  |  | **Eastings** | **Northings** |  |  |  |  |  |  | **Grass** | **Shrub** | **Tree** |
| **1** |  | 430680.256 | 347419.61 |  | Carboniferous - Bowland High Group and Craven Group |  |  | 0.70905 |  | 0.710537 | 0.710479 | 0.711881 |
|  |  |  |  |  |  |  |  |  |  | 0.000008 | 0.000008 | 0.000011 |
| **2** |  | 413998.945 | 323228.121 |  | Triassic mud-, silt-, and sandstone - Mercia Mudstone Group |  |  | 0.71142  0.71170  0.71209  0.71219  0.71239 |  | 0.710899 | 0.709836 | 0.709473 |
|  |  |  |  |  |  |  |  |  |  | 0.000010 | 0.000013 | 0.000009 |
|  |  |  |  |  |  |  |  |  |  |  |  |  |
|  |  |  |  |  |  |  |  |  |  |  |  |  |
|  |  |  |  |  |  |  |  |  |  |  |  |  |
|  |  |  |  |  |  |  |  |  |  |  |  |  |
| **3** |  | 434564.455 | 325725.42 |  | Triassic sandstone and conglomerate, interbedded – Sherwood Sandstone Group |  |  | 0.70992 |  | 0.710593 | 0.710787 | 0.711061 |
|  |  |  |  |  |  |  |  | 0.71283 |  | 0.000008 | 0.000010 | 0.000011 |
|  |  |  |  |  |  |  |  |  |  |  |  |  |
| **4** |  | 436886.361 | 325522.939 |  | Carboniferous mud-, silt-, sandstone - Millstone Grit Group |  |  | None |  | 0.711341 | 0.711178 | 0.711608 |
|  |  |  |  |  |  |  |  |  |  | 0.000008 | 0.000011 | 0.000009 |
| **5** |  | 437295.523 | 321256.869 |  | Pennine Lower Coal Measures Formation And South Wales Lower Coal Measures Formation |  |  | None |  | 0.712890 | 0.713071 | 0.712137 |
|  |  |  |  |  |  |  |  |  |  | 0.000009 | 0.000009 | 0.000010 |
| **6** |  | 443217.02 | 345471.426 |  | Pennine Middle Coal Measures Formation And South Wales Middle Coal Measures Formation |  |  | 0.7110* |  | 0.715073 | 0.714498 | 0.714916 |
|  |  |  |  |  |  |  |  | 0.7114* |  | 0.000009 | 0.000009 | 0.000008 |
| **7** |  |  | various |  | Precambrian Charnian Supergroup, Charnwood Diorites, and Ordovician Mountsorrel Igneous Complex (Johnson 2018) |  |  | 0.71098  0.71079  0.71064  0.71060  0.71110  0.71058  0.71040  0.71063  0.71039  0.71118  0.71060  0.71050  0.71047 |  | -- | -- | -- |

**SI.4) Contextual background for Repton**

The graves inside and outside the D-shaped enclosure at St. Wystan’s chapel held the skeletons of several individuals whom the excavators believed to be members of the Viking Great Army [6, 7]. Radiocarbon dating on the human remains provided results consistent with the arrival of the army in the ninth century [8]. To the west of the enclosure lay an early medieval mausoleum which contained the disarticulated charnel of at least 264 adult individuals [7, 8]. Osteological analysis determined that most of the individuals were male and this was seen as supporting evidence that these individuals, like those in the inhumation graves near the church, were part of the Viking Great Army [6, 7]. An isotopic study by Budd *et al*. [9] included the oxygen and strontium analysis of four individuals recovered from the charnel and four from the churchyard. The results were consistent with an origin in Britain (raw data available in Evans *et al.* [10] supplementary material; Montgomery *et al.* [11]). The study included only a single soil leachate from Repton, which was cautiously considered representative of the local bioavailable strontium (hereafter BASr) with a ^87^Sr/^86^Sr value of 0.7115. Since then, soil leachates have proven to be less reliable in terms of biosphere representation than other types of samples and more emphasis is put on plant sampling to investigate local BASr [3, 12]. Jarman *et al.* [13] proposed that the BASr for Repton ranged between ^87^Sr/^86^Sr 0.7112-0.7120, based on three dentine samples, one previously published soil leachate, and one faunal sample (cow).

Still, the suggestion that the enclosure at St. Wystan, Repton, was the sole base for the winter camp of the Viking Great Army is unlikely. The D-shaped earthwork at Repton encloses an area of 0.4ha, compared to the 55ha camp at Torksey [14], and 31ha at the camp at Aldwark, in North Yorkshire [15]. Recent metal-detected finds, including characteristic lead gaming pieces and Islamic coins, as well as weights, now suggest that the area known as Foremark, situated between the village of Repton and the cremation cemetery of Heath Wood, was also part of the landscape that made up the camp at Repton [16].

**SI.5) Additional contextual information on the excavation of the mounds at Heath Wood**

In each of the pre-1998 excavations, a number of mounds were described as empty – a conclusion that has now been called into question [17]. Posnansky concluded that five of the mounds excavated by him were symbolic and formed cenotaph mounds rather than actual burials, but Richards *et al.* [17] have argued that the failure to detect any remains may have been caused by the excavation strategy which could have bypassed peripherally placed token deposits.

In the most recent excavation, one of three mounds appeared to be empty (21), while Mounds 50 and 56 both contained artefacts alongside the cremated human and animal remains [17]. Mound 50 featured a sword hilt grip with a decorated silver strip (burnt), two metal studs, iron nails, and a number of molten objects, some of which may have contained silver. A hinge pivot was also present, as was a small knife, nail shanks, a clamp with rivets and clamp fragments, which were thought to have belonged to a shield rim. Animal remains included a horse, dog, sheep/goat, a possible pig, and cattle (Fig. 3)[17]. In Mound 56, a loop-headed ring-pin was recovered, which has parallels in Scandinavia, as well as in Hiberno-Norse early tenth century Scotland and Ireland, and earlier in inhumation graves on the Isle of Man [17]. A pig was also identified amongst the cremated remains. Most of the items were in poor condition upon excavation, with the exception of the ring-pin and the sword hilt grip.

**SI.6) Bone turnover considerations**

Bone remodelling is a difficult subject to do justice within the scope of this paper. Before skeletal maturity is reached and epiphyses have fused - effectively ending the growth period - most bone contains a significant portion of recent material, because bone formation rates are much higher than resorption rates during this phase (bone modelling) [18-22]. We therefore assume that the child in our study reflects strontium amassed predominantly recently during growth. Once skeletal maturity is reached, the entire process slows down and, in the absence of pathologies or adverse environmental factors, bone should maintain a relative equilibrium of resorption/formation (remodelling) on the bone surfaces to ensure bone health [19]. Modelling occurs in adults only when load or mechanics change. There is also a difference between peripheral and central skeleton in terms of cortical and trabecular turnover rates, and bone surfaces adjacent to red marrow appear to remodel faster [19]. According to clinical literature, the cortical bone of the rib turns over ‘twice as fast’ as that of other cortical bone [23] (see pages 35-36), something that has also been articulated by Parfitt [19] in his observation of the different rate of turnover between central and peripheral skeleton. According to Frost [24], bone formation (note: not the remodelling rate) in the cortical bone of the 6th rib of an adult should be around 2-6% per year, but Frost’s data excludes activity in the periosteal and endosteal surfaces. It is certain that the overall turnover rate is therefore generally higher than this, because most remodelling occurs on these surfaces [19]. Kerley [20] have shown that remodelling in the midshaft cortex of the femur, tibia, and fibula of adults occur to a slower degree with a rate of 3%, 2.5%, and 4% respectively [23] (page 34). There are problems with generalising these rates and applying them to all adult individuals, but in the absence of observable pathologies we accept these observed trends for the interpretation of our data.

# **References**

1. Evans JA, Montgomery J, Wildman G, Boulton N. Spatial variations in biosphere 87Sr/86Sr in Britain. Journal of the Geological Society. 2010;167(1):1-4.

2. Evans JA, Chenery CA, Mee K, Cartwright CE, Lee KA, Marchant AP, et al. Biosphere Isotope Domains GB (V1): Interactive Website: British Geological Survey; 2018.

3. Snoeck C, Ryan S, Pouncett J, Pellegrini M, Claeys P, Wainwright AN, et al. Towards a biologically available strontium isotope baseline for Ireland. Science of The Total Environment. 2020;712:136248. doi: 10.1016/j.scitotenv.2019.136248.

4. Johnson LJ. Finding radiogenic Sr-isotope biospheres: can a home in Britain be found for people with high 87Sr/86Sr? . unpublished: Durham University; 2018.

5. Claisse P, Calla C, editors. Rail ballast: conclusions from a historical perspective. Proceedings of the Institution of Civil Engineers-Transport; 2006: Thomas Telford Ltd.

6. Biddle M, Kjølbye-Biddle B. Repton and the Vikings. Antiquity. 1992;66(250):36-51. doi: 10.1017/s0003598x00081023.

7. Biddle M, Kjølbye-Biddle B. Repton and the ‘great heathen army’, 873–4. In: Graham-Campbell J, editor. Vikings and the Danelaw: select papers from the Proceedings of theThirteenth Viking Congress, Nottingham and York, 21–30 August 1997. Oxford: Oxbow; 2001. p. 45-96.

8. Jarman CL, Biddle M, Higham T, Bronk Ramsey C. The Viking Great Army in England: new dates from the Repton charnel. Antiquity. 2018;92(361):183-99. doi: 10.15184/aqy.2017.196.

9. Budd P, Millard A, Chenery C, Lucy S, Roberts C. Investigating population movement by stable isotope analysis: a report from Britain. Antiquity. 2004;78(299):127-41. doi: 10.1017/s0003598x0009298x.

10. Evans JA, Chenery CA, Montgomery J. A summary of strontium and oxygen isotope variation in archaeological human tooth enamel excavated from Britain. Journal of Analytical Atomic Spectrometry. 2012;27(5):754-64. doi: 10.1039/c2ja10362a. PubMed PMID: WOS:000302755200004.

11. Montgomery J, Grimes V, Buckberry J, Evans JA, Richards MP, Barrett JH. Finding Vikings with isotope analysis: The view from wet and windy islands. Journal of the North Atlantic. 2014;7:54-70.

12. Ryan SE, Snoeck C, Crowley QG, Babechuk MG. ^87^Sr/^86^Sr and trace element mapping of geosphere-hydrosphere-biosphere interactions: A case study in Ireland. Applied Geochemistry. 2018;92:209-24. doi: 10.1016/j.apgeochem.2018.01.007. PubMed PMID: WOS:000432195100019.

13. Jarman CL, Biddle M, Fullagar P, Horton M. Viking Age Repton: Strontium evidence for the mobility and identity of the charnel dead. Church Archaeology. 2019;19:73-90. doi: 10.5284/1081986.

14. Hadley DM, Richards JD. The winter camp of the Viking Great Army, AD 872–3, Torksey, Lincolnshire. The Antiquaries Journal. 2016;96:23-67. doi: 10.1017/s0003581516000718.

15. Williams G. A Riverine Site Near York : A Possible Viking Camp? Williams G, editor. London: British Museum Press; 2020. 160 p.

16. Jarman CL. River Kings: A New History of Vikings from Scandinavia to the Silk Road: HarperCollins Publishers; 2021.

17. Richards JD, Beswick P, Bond J, Jecock M, McKinley J, Rowland S, et al. Excavations at the Viking barrow cemetery at Heath Wood, Ingleby, Derbyshire. The Antiquaries Journal. 2004;84:23-117.

18. Parfitt AM. What is the normal rate of bone remodeling? Bone. 2004;35(1):1-3. doi: 10.1016/j.bone.2004.03.022. PubMed PMID: WOS:000222494100001.

19. Parfitt AM. Chapter 36 - Skeletal Heterogeneity and the Purposes of Bone Remodeling: Implications for the Understanding of Osteoporosis. In: Marcus R, Feldman D, Dempster DW, Luckey M, Cauley JA, editors. Osteoporosis (Fourth Edition). San Diego: Academic Press; 2013. p. 855-72.

20. Kerley ER. The microscopic determination of age in human bone. American Journal of Biological Anthropology. 1965;23(2):149-63. doi: <https://doi.org/10.1002/ajpa.1330230215>.

21. Kenkre J, Bassett J. The bone remodelling cycle. Annals of Clinical Biochemistry: International Journal of Laboratory Medicine. 2018;55(3):308-27. doi: 10.1177/0004563218759371.

22. Hill PA, Orth M. Bone remodelling. Br J Orthod. 1998;25(2):101-7. doi: 10.1093/ortho/25.2.101. PubMed Central PMCID: PMC9668992.

23. ICRP. Alkaline Earth Metabolism in Adult Man. Oxford: The International Commission on Radiological Protection, 1973.

24. Frost HM. Tetracyclene-based Histological analysis of Bone Remodeling. Calcified Tissue Research. 1969;3(3):211-&. doi: 10.1007/bf02058664. PubMed PMID: WOS:A1969D435400001.
